# The *SKP1-Like* Gene Family of Arabidopsis Exhibits a High Degree of Differential Gene Expression and Gene Product Interaction during Development

**DOI:** 10.1371/journal.pone.0050984

**Published:** 2012-11-30

**Authors:** Mohammad H. Dezfulian, Danielle M. Soulliere, Rajdeep K. Dhaliwal, Madhulika Sareen, William L. Crosby

**Affiliations:** Department of Biological Sciences, University of Windsor, Windsor, Ontario, Canada; Iowa State University, United States of America

## Abstract

The *Arabidopsis thaliana* genome encodes several families of polypeptides that are known or predicted to participate in the formation of the SCF-class of E3-ubiquitin ligase complexes. One such gene family encodes the Skp1-like class of polypeptide subunits, where 21 genes have been identified and are known to be expressed in Arabidopsis. Phylogenetic analysis based on deduced polypeptide sequence organizes the family of ASK proteins into 7 clades. The complexity of the *ASK* gene family, together with the close structural similarity among its members raises the prospect of significant functional redundancy among select paralogs. We have assessed the potential for functional redundancy within the *ASK* gene family by analyzing an expanded set of criteria that define redundancy with higher resolution. The criteria used include quantitative expression of locus-specific transcripts using qRT-PCR, assessment of the sub-cellular localization of individual ASK:YFP auto-fluorescent fusion proteins expressed *in vivo* as well as the *in planta* assessment of individual ASK-F-Box protein interactions using bimolecular fluorescent complementation techniques in combination with confocal imagery in live cells. The results indicate significant functional divergence of steady state transcript abundance and protein-protein interaction specificity involving ASK proteins in a pattern that is poorly predicted by sequence-based phylogeny. The information emerging from this and related studies will prove important for defining the functional intersection of expression, localization and gene product interaction that better predicts the formation of discrete SCF complexes, as a prelude to investigating their molecular mode of action.

## Introduction

Genetic and molecular studies in the model plant species *Arabidopsis thaliana* have emphasized the importance of ubiquitin-mediated targeted protein degradation for the regulation of diverse plant-specific processes [1]–[3]. Genetic surveys for the identification of loci that regulate patterning and development have revealed numerous genes that encode known or predicted subunit components of both RING and HECT classes of E3-ubiquitin ligases (E3-Ub). Functional analysis of mutants at many of these loci suggests a central role for post-translational protein degradation in such plant-specific functions as auxin response [4], [5], response to jasmonate [6], maintenance of circadian rhythm [7], [8], photomorphogenesis [9] and floral development [10], to name but a few.

Arabidopsis is an attractive model system in which to study the role of post-translational protein modification in the regulation of development, in part due to its many technical advantages coupled with a wealth of genetic resources and genomic data sets that facilitate hypothesis generation and functional analysis. Although Arabidopsis possesses one of the smallest known angiosperm genomes studied to date, with 147 Mbp encoding approximately 27,000 protein coding genes, it has nevertheless been annotated to contain over 1,500 genes that are known or predicted to encode subunits of ubiquitin ligase complexes (nearly 6% of the coding capacity) including more than 700 F-box proteins comprising about 3% of the Arabidopsis genome coding capacity [1]. This genetic complexity and allocation of gene coding capacity to SCF-ligase complexes involved in post-translational protein turnover-related processes is prominent in plants, and can be compared with that of *Homo sapiens* where only 69 F-Box genes have been identified [11].

The quaternary subunit composition of the SCF-class of E3-Ub complexes have been studied in numerous model systems [5], [12]–[15], and are minimally comprised of one each of the F-Box, Skp1-like, Rbx1 and Cul1 class of polypeptide subunits [15], [16]. For the most part, the subunit stoichiometry of individual functional complexes has yet to be experimentally determined, although crystal structures for select SCF-class E3-ubiquitin ligases have been described for yeast, human and Arabidopsis protein complexes [5], [13], [15], [17]. Recent evidence suggests that assembly of SCF-class E3-Ub ligase complexes may result in protein interactions involving multiple F-Box subunits [16], [18]–[20]. The characterization of subunit interaction potential and stoichiometry for select SCF-class of E3-Ub complexes will likely prove important for understanding the combinatorial diversity of complexes that may form across developmental time and space, together with a determination of their associated biological function [21]. Given the relatively large number of genes that are known or predicted to function in post-translational ubiquitination of proteins in Arabidopsis, together with the close primary structural similarity of the genes within the *ASK* gene family, a significant degree of functional redundancy might be expected. Knock down-based functional assessment of potentially redundant genes is commonly conducted on the basis of deduced amino acid sequence similarity, often ignoring functional aspects of genetic redundancy such as expression, localization and gene-product interaction. Since amino acid sequence similarity is not the only factor contributing to gene redundancy, such studies often result in a lack of observable phenotype. Therefore, a more insightful assessment of gene redundancy potential within the *ASK* gene family should include multiple aspects of gene function as a foundation upon which any hypothesis driven functional assessment might be derived.

Among the four canonical subunit classes that are known to participate in the formation of the SCF-class of E3-Ub complexes, relatively little is known about the functional role of the individual **A**rabidopsis **Sk**p1-like family of polypeptides (ASK proteins). The gene family encoding ASK polypeptides in Arabidopsis is complex, with 21 known expressed genes compared to a single Skp1-like gene in yeast and *H. sapiens* [22]–[24]. Phylogenetic analysis of the *ASK* gene family based upon the deduced amino acid sequence has been used to define clades that are suggested to predict functional redundancy among individual *ASK* genes [24]. Indeed, studies involving loss of function alleles in the closely related genes *ASK1* and *ASK2* indicate these two genes perform strongly overlapping functions that are essential for early patterning and development [24]. These studies are complemented by the finding that the ASK1 and ASK2 proteins interact broadly with F-Box proteins, suggesting correspondingly diverse roles in the formation of functional SCF complexes in Arabidopsis [21]. The function of the remaining 19 members of the *ASK* gene family remains largely uncharacterized.

Expression studies involving *ASK* genes in Arabidopsis have generally involved reporter-gene fusion and non-quantitative RT-PCR approaches, where such studies indicate that certain members of the *ASK* gene family are preferentially expressed in a subset of organs and tissues at different times during development [23], [25]. Quantitative studies provide the desired precision needed for higher-resolution comparison of *ASK* gene expression and clustering on the basis of expression - an aspect that has been largely neglected from previous studies to date. This type of information can also be important for defining the extent of expression overlap among SCF subunits, and for elucidating their potential to participate in the formation of SCF complexes.

In this report we analyze functional redundancy within the *ASK* gene family by evaluating three aspects of ASK protein and cognate gene redundancy; a quantitative analysis of *ASK* transcript abundance in select organs during development; the sub-cellular compartmentation of expressed tagged ASK proteins; and an *in situ* analysis using bimolecular fluorescence complementation (BiFC)-based approaches to assess protein-protein interactions involving ASK polypeptides. Taken together, the study concludes that phylogenetic relatedness based upon primary amino acid sequence similarity is a poor predictor of redundancy defined by steady-state transcript abundance, sub-cellular localization and gene product interactions exhibited by products of the *ASK* gene family. The results are discussed in the context of the potential for combinatorial complexity of SCF complex formation by products of the *ASK* gene family.

## Materials and Methods

### Plant Material and Growth Conditions

Arabidopsis ecotype Columbia (Col-0) was used throughout the study (ABRC stock No. CS7000). Seeds were sterilized by brief sequential washes in 50% ethanol and 50% Bleach/SDS prior to plating on 0.5X MS medium [26] containing 1% sucrose, and 0.8% agar. Seeds from treated plants were harvested, sterilized and stratified at 4°C for 2–3 days prior to plating and germination on solid 0.5X MS medium containing 50 µM D,L-phosphinothricin (a gift from Bayer Crop Science Canada) using the rapid procedure as described [27]. Two to three independent transgenic plants were subsequently selected for each analysis.

### Plasmid Construction

For all expression constructs, a summary of standard gene nomenclature and molecular constructs are summarized in Table S1. For *35S-YFP-ASK(s)* plasmid assembly, Gateway™-compatible cDNA clones were obtained from the ABRC stock center (Columbus, OH; see Table S1) and were cloned into pEarleyGate104 [28] using the commercial Gateway™ recombination system (Invitrogen). To assemble *35S-ASK1-CFP, 35S-TIR1-CFP* and *35S-CUL1-CFP* constructs, termination codons were first removed from the cDNA clones by PCR amplification of the coding region using the indicated primers (Table S2). Amplified PCR products were subsequently cloned into pDONR221 using the Gateway™ recombination system prior to recombination into pEarleyGate102 [28]. All recombinants were sequenced to verify the integrity of expression constructs.

To assemble the described BiFC constructs, cDNA domains coding for the indicated ASK and F-Box proteins were fused to the N- or C-terminal domains of EYFP (nEYFP or cEYFP). The *ASK* Gateway™-based cDNA clones (listed in Table S3) were subsequently cloned into BiFP2 [29] vectors using the Gateway™ recombination system, resulting in an experimental set of *35S-cEYFP-ASK* constructs. Similarly, F-Box protein-encoding cDNA clones were cloned into BiFP3 [29], resulting in the *35S-nEYFP-F-Box* set of constructs. A complete list of the vectors used is summarized in Table S4. In those instances where the entry clone and destination plasmid backbone carried the same selection marker, for cloning purposes 1 µg of the pEntry plasmid was initially digested and counter-selected by restriction digestion with MluI prior to input to the LR clonase reaction. Correct expression of the desired BiFC fusion products was independently evaluated in *N. benthamiana* leaves by construction, transformation and expression of select ASK and F-Box fusions in the pE-SPYNE-GW and pE-SPYCE-GW gateway compatible BiFC vectors harboring Myc and HA tags at the N-terminus of the split YFP domain, respectively [30].

### Generation of Transgenic Plants

The *35S-YFP-ASK* constructs were transformed into *Agrobacterium tumefaciens* strain AGL1 by electroporation, and the presence of transgenes were confirmed by *in situ* PCR. Plant transformations were performed using the floral dip method [31]. Seeds from treated plants were harvested, sterilized and stratified at 4°C for 2–3 days prior to plating and germination on solid 0.5X MS medium containing 50 µM D,L-phosphinothricin (a gift from Bayer Crop Science Canada) using a rapid procedure previously described [27]. Two to three independent transgenic plants were subsequently selected for each analysis.

### Western Blotting

Protein was extracted following fusion construct expression in a *N. benthamiana* transient expression system.100 mg of leaves, infiltrated with the indicated expression constructs were ground in liquid nitrogen and mixed with 100 µl of extraction buffer [100 mm Tris·HCl (pH 7.5), 150 mM NaCl, 5 mM EDTA, 10 mM 2-mercaptoethanol, 10% glycerol, 0.1% Triton X-100, 1× EDTA free Complete protease inhibitors (Roche Applied Science)] [32]. Extracts were centrifuged at 14,000 *g* for 15 min and the supernatant was collected. Proteins were resolved by 12% SDS-PAGE and transferred to a PVDF membrane using a TransblotSD™ Semi Dry Transfer (Biorad) in Bjerrum and Schaefer-Nielsen buffer (48 mM TRIS, 39 mM glycine, 20% methanol). The blots were incubated with either anti-HA (Y-11) or anti-*c-myc* (9E10) antibodies (Santa Cruz Biotechnology) at a 1∶1,000 dilution in 1% skim milk powder in TBST overnight at 4°C. Blots were washed 3 times with TBST for 10 min and incubated with secondary antibody at a 1∶10,000 dilution in 2% skim milk/TBST for 1 h at room temperature. Blots were subsequently washed and exposed using Pico West Reagent™ (Fisher Scientific) and imaged using an AlphaImager device (Alpha Innotech Corp., San Leandro, CA).

### Transient Expression in *N. benthamiana*

All BiFC vectors were transformed into *Agrobacterium tumefaciens* strain AGL1 by electroporation, and the presence of transgenes was confirmed by *in situ* PCR. Mixed Agrobacterium cultures were co-infiltrated to the abaxial surface of 3–4 week-old *N. benthamiana* plants as described [33]. The p19 protein of tomato bushy stunt virus was co-expressed with all binary BiFC expression constructs in order to suppress gene silencing [33]. Fluorescence signals were visualized in epidermal cell layers of the leaves after 3 days of infiltration using confocal microscopy.

### Confocal Imaging

Imaging of BiFC signals *in planta* was done using an Olympus Model FV1000 point-scanning/point-detection laser scanning confocal microscope. Cyan fluorescent protein (CFP), yellow fluorescent protein (YFP) and propidium iodide (PI) were excited by using 440, 512 and 543 nm laser lines, respectively. When using multiple fluorophores simultaneously, images were acquired sequentially in order to reduce excitation and emission overlaps. Olympus water immersion PLAPO60XWLSM (NA 1.0) and UPLSAPO 20× (NA 0.75) objectives were employed. Image acquisition was conducted at a resolution of 512×512 pixels, with a scan rate of 10 ms per pixel. Olympus FLUOVIEW v1.5 software was used for image acquisition and the export of TIFF files. Figures were assembled using GIMP 2.0 (http://www.gimp.org/).

### Quantitative Real Time PCR

Total RNA was isolated using a commercial mini-preparation kit (RNeasy™, Qiagen) and contaminating DNA was removed using an immobilized DNAse column (RNAse-Free DNAse Set™, Qiagen). Two micrograms of total RNA was used as template for first strand cDNA synthesis in a 20 µL reaction using the RevertAid™ synthesis kit (Fermentas). The resulting cDNA was diluted 1∶20 and 1.5 µL of cDNA was used in a standard 20 µL PCR reaction. Analysis of gene expression used the Maxima™ Sybr-green qPCR master mix (Fermentas) in an Applied Biosystems 7300 RT- PCR System, following the manufacturer’s instructions. The primers used in the qRT-PCR analyses are listed in Table S4. To identify the optimal internal reference control *β6-tubulin*, *ACTIN2* and *UBQ10* expression was assessed across all organs. *ACTIN2* (At3g18780) transcript expression showed the least amount of variation across all tissues examined and was used as the internal standard in order to normalize the qRT-PCR data. Real time PCR results were analyzed using Q-Gene software that expresses data as Mean Normalized Expression (MNE) [34] which is directly proportional to the amount of mRNA of the target genes relative to the amount of mRNA of the *ACTIN2* internal reference standard. In brief, to calculate MNE, the PCR efficiency (E), mean cycle threshold (Ct) and related standard errors (SEs) were used to calculate ECt for both the reference and target genes. MNE was subsequently calculated by dividing the ECt of the reference gene by the ECt of the target gene. Gene expression is depicted in MNE units after *ACTIN2* normalization (Table S5). Two biological and 3 sample replicates were performed for each tissue/organ studied while taking into consideration the efficiency of the reaction for each primer combination [34]. Data is depicted as the mean of two independent biological repetitions ± SE. To calculate the relative fold-change expression among *ASK* genes, the ‘Relative Expression Software Tool-Multiple Condition Solver’ (REST-MCS) was used [35], [36]. Pearson-based hierarchical clustering of *ASK* gene expression data was done using the online version of Expression Profiler found at EBI (http://www.ebi.ac.uk/expressionprofiler/) [37].

### Phylogenetic Analysis

ASK protein sequences were retrieved from the TAIR10 genome annotation data set (http://www.arabidopsis.org/). Multiple alignments were carried out using the CLUSTAL algorithm found within the MEGA 4 software suite [38] using a BLOSUM 30 matrix with a gap penalty of 10, an extended gap penalty of 0.2 and a gap distance of 5. The tree was constructed using the Neighbor Joining (NJ) method utilizing the p-distance substitution method in MEGA 4 and node reliability was calculated using 1,000 Bootstrap replicas [38]. In order to evaluate different clustering methods for their possible effect on tree construction, we compared three different methods: NJ Maximum Parsimony, Un-weighted Pair Group and Arithmetic Mean (UPGMA) using MEGA 4. This comparison revealed slight differences in the clustering pattern. Clustering using UPGMA resulted in the grouping of ASK13 with ASK11 and ASK12 but not with ASK5 and ASK6. However, based on the chromosomal location and supporting literature [39] we believe that *ASK13* is a result of tandem duplication of *ASK5,* thus NJ clustering was used for this study.

## Results

**Figure 1:**
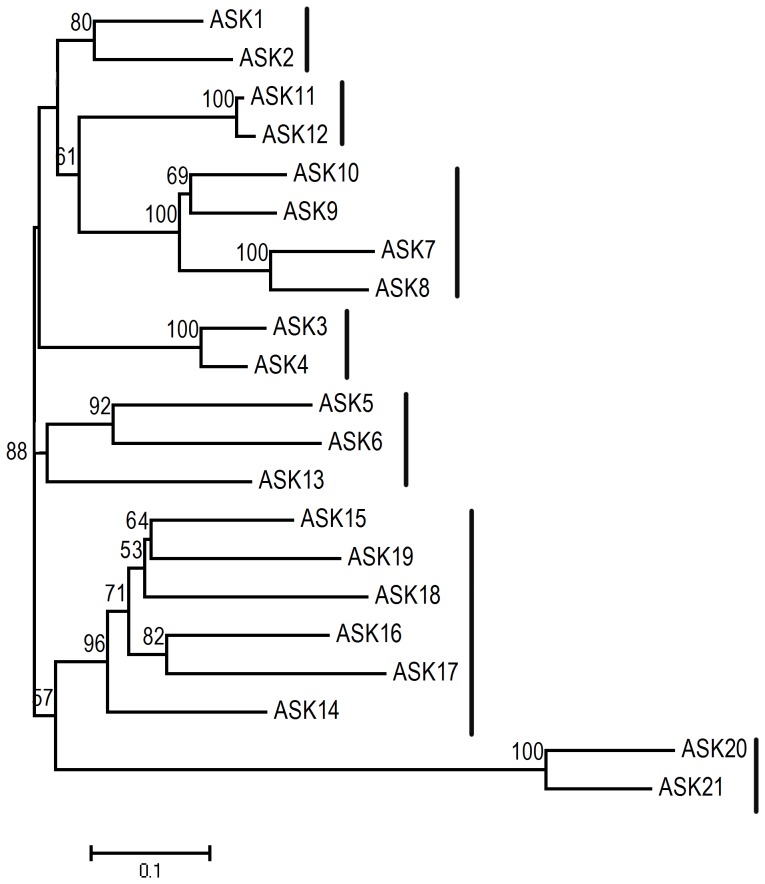
Relationship of the *ASK* gene family in Arabidopsis. The phylogenetic grouping of *ASK* genes based on their deduced primary amino acid sequence was calculated using the NJ method described. The genes are grouped into seven distinct clades as denoted by the vertical lines. Numbers at the branches represents percentage bootstrap support calculated for a 1000 replicates. All tree branches are scaled to the number of amino acid substations per site.

A phylogenetic analysis of relatedness within the *ASK* gene family of Arabidopsis reveals that the 21 members of this gene family fall into 7 distinct clades when clustered on the basis of primary deduced amino acid sequence, as summarized in Fig. 1. This clustering of the predicted ASK proteins is consistent with published results, where *ASK1* and *ASK2* are grouped within a clade and were found to overlap functionally and are known to be essential for early development in Arabidopsis [40]. We used this phylogenetic tree as a basis to assess the functional relatedness among *ASK* paralogs as measured by three functional criteria: tissue/organ-specific transcript abundance, sub-cellular localization of YFP-tagged ASK proteins, and the protein-protein interaction profiles of ASK proteins in conjunction with selected F-Box proteins expressed as BiFC fusion constructs.

### qRT-PCR Analysis of *ASK* Gene Transcript Abundance

**Figure 2:**
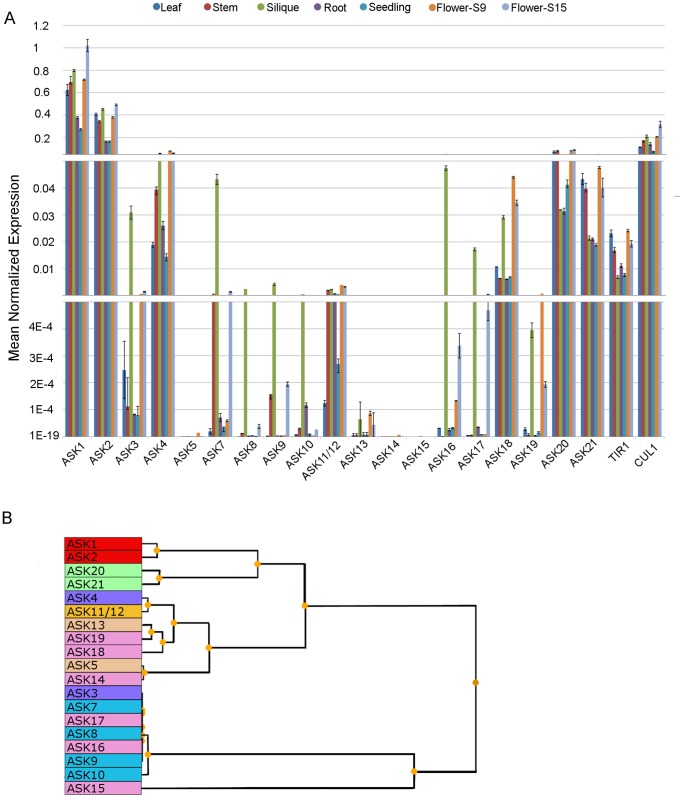
Real time expression analysis of the Arabidopsis ASK gene family. A ; RNA from the indicated organs was isolated and used to assess ASK gene expression as described in the methods. Organs examined were 4–5-day-old seedlings, stage 5 leaves, green stem, green siliques, stage 9 flowers, and stage 15 flowers. Gene expression is depicted in MNE units ± SE after *ACTIN2* normalization. Data are means of two independent biological repetitions ± SE. **B**; Cluster summary of the Pearson-based hierarchical clustering of *ASK* gene expression among select organs. The colors represent genes within the different phylogenetic clades as defined within Fig 1.

A qRT-PCR analysis of transcript abundance was undertaken for members of the *ASK* gene family which reside in common phylogenetic clades based on their primary deduced amino acid sequence (*ASK1,2,3,4,5,6,7,8,9,10,11/12,13,14,15,16,17,18,19,20,21*). Included were two genes known to be associated with SCF type E3-Ub function (*CUL1*, *TIR1*) as well as *β6-tubulin*, *Actin2* and *UBQ10* as internal reference controls. We were unable to design unique qRT-PCR primers capable of distinguishing *ASK11* from *ASK12* transcripts, due to the high DNA sequence similarity (99.3%) between these two genes. Thus, for purposes of this study, a single primer pair for *ASK11/ASK12* was used. Transcript-specific primers and their associated genomic identifiers are listed in Tables S1 and S2. Transcript abundance was quantified in cDNA preparations constructed from total RNA fractions prepared from rosette leaves of plants prior to stage 5.2, roots of 7-day-old seedlings, green stems (1^st^ and 2^nd^ internodes of bolted plants), green siliques with seeds (late heart to mid torpedo embryo), whole 5-day-old seedlings, old stage 9 whole flowers (petal primordia stalked at base) from 19–23-day-old plants and stage 15 whole flowers (stigma extends above long anther) from 21–23-day-old plants [41]. Quantitative RT-PCR analysis and cDNA measurements were the result of 3 independent experiments involving 2 biological replicate samples for each as summarized in Fig. 2A, where data for each gene/organ combination are shown and clustered on the basis of their relative expression abundance across all biological samples analyzed (Fig. 2B).

**Figure 3:**
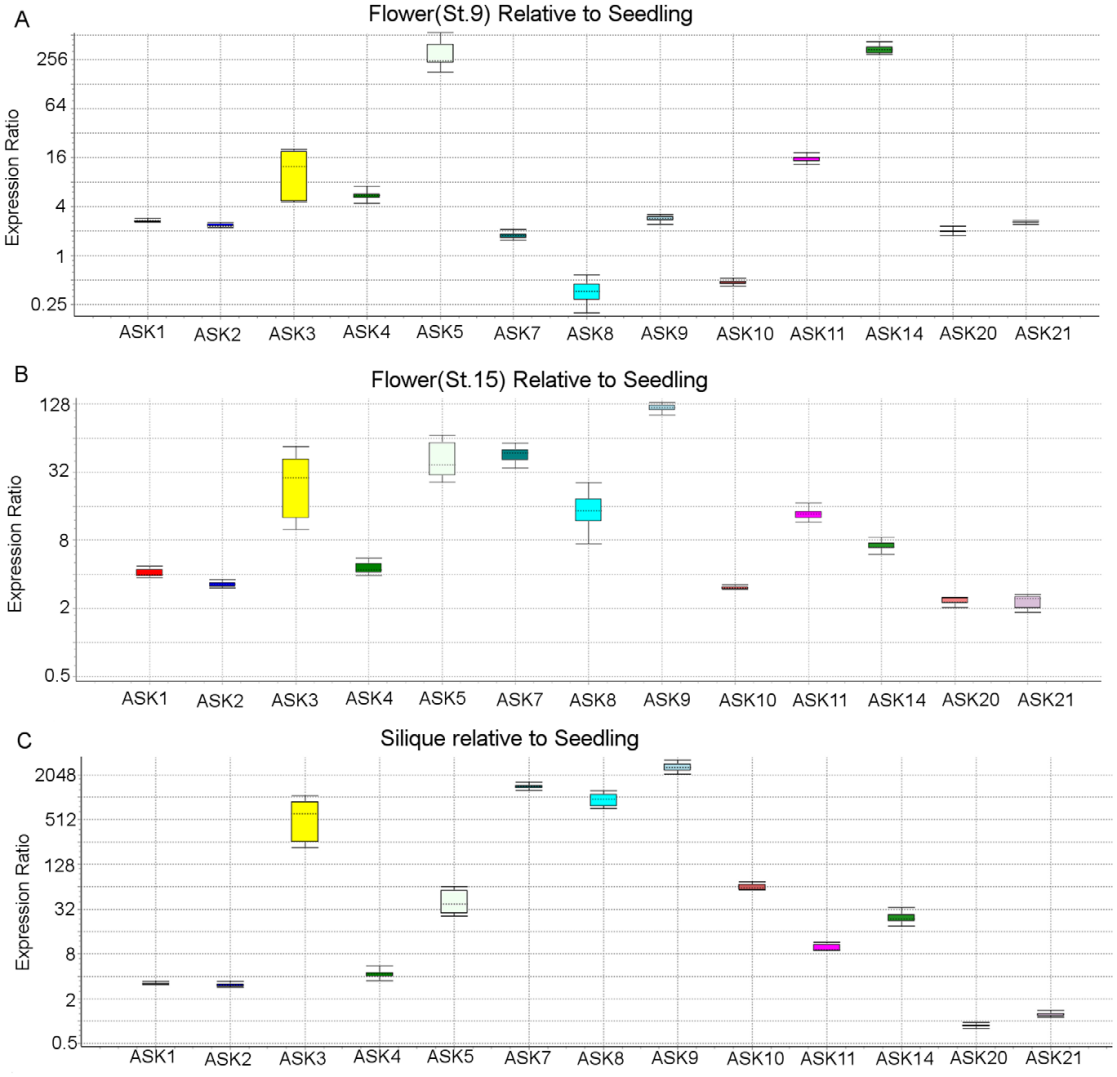
Relative organ-specific abundance of cDNAs for select ASK genes. All gene expression was normalized relative to *ACTIN2* expression. **A**; Relative abundance of *ASK* gene cDNAs in flowers from stage 9 plants relative to seedlings. **B**; Relative abundance of *ASK* cDNAs in stage 15 flowers relative to seedlings. **C**; Relative expression of *ASK* genes in siliques relative to seedlings.

From this analysis, and in agreement with previously published studies, the relative abundance of mRNAs for *ASK1* and *ASK2* were found to be elevated and constant in comparison to other *ASK* genes across all biological organs examined [23], [42]. However for the common clade comprised of *ASK3* and *ASK4*, the transcripts for these two genes were found to be expressed at markedly different steady state levels, where the abundance of *ASK4* mRNA was consistently an order of magnitude higher in all organs examined except siliques. Moreover, the relative expression of *ASK3* was found to be elevated in reproductive organs in comparison to other organs, whereas *ASK4* mRNA abundance was relatively constant across all organs examined. When expressed as the ratio relative to whole seedling organs, *ASK3* mRNA was found to be 500-fold more abundant in green siliques (Fig. 3C) and was elevated in stage 9 and stage 15 flowers excised from 21–25-day-old plants (Fig. 3A,B). In contrast, the expression ratio for *ASK4* mRNA was found to be only modestly elevated (about 3-4-fold) in these same organs.

A distinct expression pattern was observed within the clade comprised of *ASK5/6/13* where *ASK13* transcripts were found to have a relatively higher abundance in foliar tissues and the silique. *ASK6* transcripts were not detectable in the organs examined (data not shown), whereas its close paralog *ASK5* albeit lower than *ASK13*, expressed measurable transcripts in all organs examined (Fig. 2A). Although *ASK6* has been suggested in the literature to be a pseudogene [43] we think this is unlikely since *ASK6* cDNA has been enriched from a low-abundance RNA library and the corresponding clone is available from the ABRC stock center (stock number PENTR221-AT3G53060).

Within the phylogenetic clade defined by *ASK7,8,9,10*, all four gene transcripts were found to be relatively abundant in tissues from later-stage flowers (stage 15) and siliques, whereas their abundance was relatively low in earlier (stage 9) flowers compared to seedlings (Fig. 3A,B,C). The *ASK10* gene expression pattern was unique among genes defining the *ASK7,8,9,10* clade in that it exhibited a conspicuously lower relative transcript level compared to the other 3 genes in the clade.

For the clade defined by *ASK20* and *ASK21*, transcripts were expressed at a relatively low and constant level, with a modest increase in abundance (2- to 4-fold) in stage 9 and stage 15 flowers (Fig. 3A,B,C). Interestingly, the expression pattern of *ASK20* and *ASK21* most closely resembled that of *ASK1* and *ASK2* in light of their constant level of expression throughout development of Arabidopsis. The lowest transcript level in comparison to all other *ASK* genes studied was observed for *ASK14,15* which co-reside within the clade comprising *ASK14,15,16,17,18,19.* However, transcripts for these two genes showed a distinct expression pattern between them in that *ASK15* was expressed only in roots and siliques (Fig. 2A) whereas *ASK14*, similar to *ASK5*, was expressed preferentially in early stages of flower development (stage 9) followed by a decline in transcript abundance at later stages of flower maturation (stage15) and silique development.

The data underlying the observed pattern of relative abundance of mRNAs for each *ASK* gene, across all organ sources examined, was submitted to clustering analysis with the resulting grouping presented in Fig. 2B. The data indicate that *ASK4* and *ASK 11/12* are similar with respect to the relative abundance of their respective transcripts and their pattern of expression across the organs examined. In this analysis, *ASK3* mRNA abundance in the organ samples assessed was distinct from *ASK4* as its closest primary sequence paralog in Arabidopsis.

It should be noted that several deviations were observed between the expression data here versus the microarray data available through several databases such as Bio-Array [44], [45] and GeneInvestigator™ [46]. In those instances where deviations were observed, such variations could be attributed to the probes used in the microarray construction. For instance, and in contrast to our findings, closely related genes such as *ASK3* and *ASK4* as well as *ASK20* and *ASK21* exhibit identical expression behaviour when measured using microarray approaches (Figure S3), where the results can be explained by the use of probes that did not distinguish between the mRNAs encoded by these two genes.

### Sub-cellular Localization of YFP:ASK Fusion Proteins in Arabidopsis

**Figure 4:**
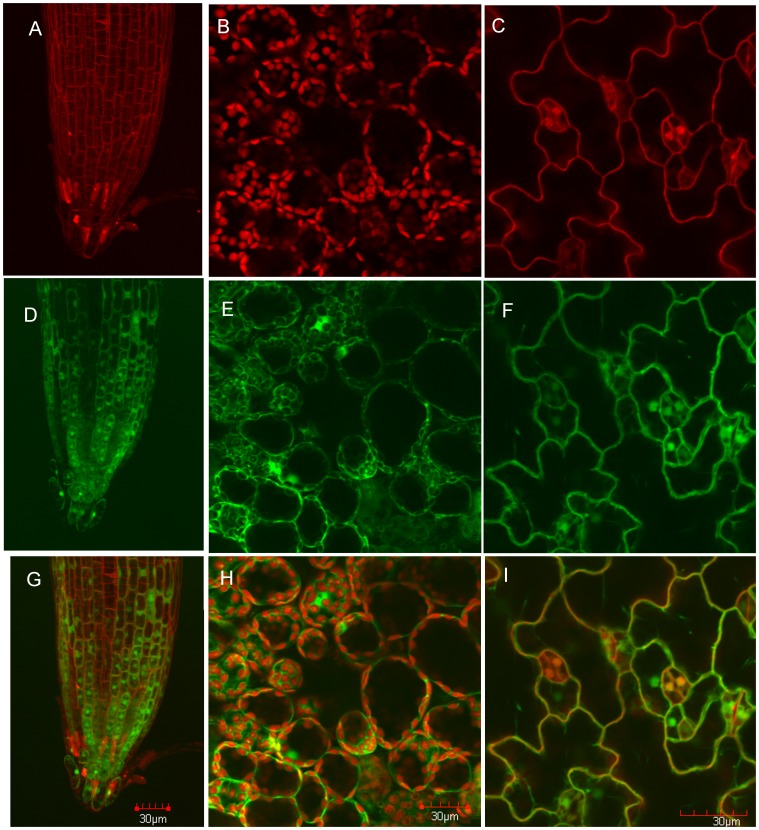
Confocal imaging and sub-cellular localization of ASK proteins in transgenic Arabidopsis. A&C ; Propidium iodide-stained epidermal root cell walls or leaf epidermal cell walls and guard cell nuclei. **B**; Chlorophyll auto-fluorescence from the leaf mesophyll cell layer. **D–F**; stable expression of N-terminal YFP-tagged ASK3 protein in transgenic Arabidopsis. **G–I**; merged channels corresponding to panels (**A–C**) and (**D–F**).

One explanation for the presence of the large number of ASK proteins in Arabidopsis could be a corresponding diversity of sub-cellular localization for some of the ASK proteins. A study was therefore undertaken to evaluate the sub-cellular localization profile of 9 selected ASK proteins expressed as functional fusions with the YFP auto-fluorescent protein in transgenic Arabidopsis. The YFP:ASK fusion proteins were expressed from CaMV 35S promoter constructs assembled and transformed into Arabidopsis as described, and the results are summarized in Fig. 4. Propidium iodide (PI) staining was used to visualize cell walls and general morphology in whole mounts of roots and 5-day-old seedling leaf epidermal cells including guard cells (Fig. 4A,C). The signal arising from chlorophyll auto-fluorescence is shown in Fig. 4B. In parallel, cellular expression and sub-cellular localization of the YFP:ASK3 fusion protein is shown in Fig. 4D–F, and the composite PI-YFP images is shown in Fig. 4G–I. Taken together, and in agreement with previous studies described for *ASK1* [47], [48], YFP:ASK3 proteins accumulate in both the nucleus and the cytoplasm in epidermal leaf cells of Arabidopsis. We observed no overlap between the YFP and chlorophyll auto-fluorescence signals, suggesting that the extra-nuclear YFP:ASK3 fusion protein does not detectably localize to the chloroplast and largely accumulates in the cytoplasm.

This same pattern of YFP fusion protein accumulation in the nucleus and cytoplasm, with no significant localization to chloroplasts was reiterated across all other YFP:ASK gene fusions assessed (*ASK1,2,3,4,5,6,8, 9,10*) with the exception of *ASK8* (see Figure S5). The YFP:ASK8 protein fusion, when expressed in transgenic Arabidopsis was found to apparently aggregate predominantly in the nuclei of epidermal leaf cells, resulting in highly localized and intense YFP signals. Notably, these aggregates were not observed in root tissues of the same transgenic lines (Figure S4A). In order to investigate whether the apparent aggregation of ASK8 was the result of YFP:ASK8 over-expression, the expression of this fusion was compared to that of the YFP:ASK1 protein using Western blot analysis. As shown in Figure S4B, the level of YFP:ASK8 expression in three independent lines was qualitatively lower than that of the YFP:ASK1 fusion protein. Although ASK sub-cellular localization resembles that of GFP sub-cellular localization, the lack of available ASK protein-specific antibodies precludes the possibility of taking immunohistochemical approaches to localizing nascent ASK proteins *in situ.* However, we noted that the majority of reported SCF substrates in Arabidopsis are known transcription factors [5], [49], which is consistent with our observation of a general nuclear localization of YFP-ASK fusion proteins. To investigate whether N-terminal fusions could mask nascent localization signals, C-terminal CFP fusion constructs using the *ASK1* coding region were generated, and a similar localization pattern was observed following transient expression in *N. benthamiana* leaves (Figure S2C). In addition, we asked whether other known SCF ligase subunits exhibited a similar localization pattern to ASK proteins, suggesting the potential for co-localization and participation in SCF complex assembly. Upon assessing the localization pattern of TIR1 and CUL1 as C-terminal CFP fusions, we found that a similar localization was observed relative to the YFP:ASK protein studies described above (Figure S2A,B).

### ASK-F-Box Protein Interaction Profiling *In Vivo*

**Figure 5:**
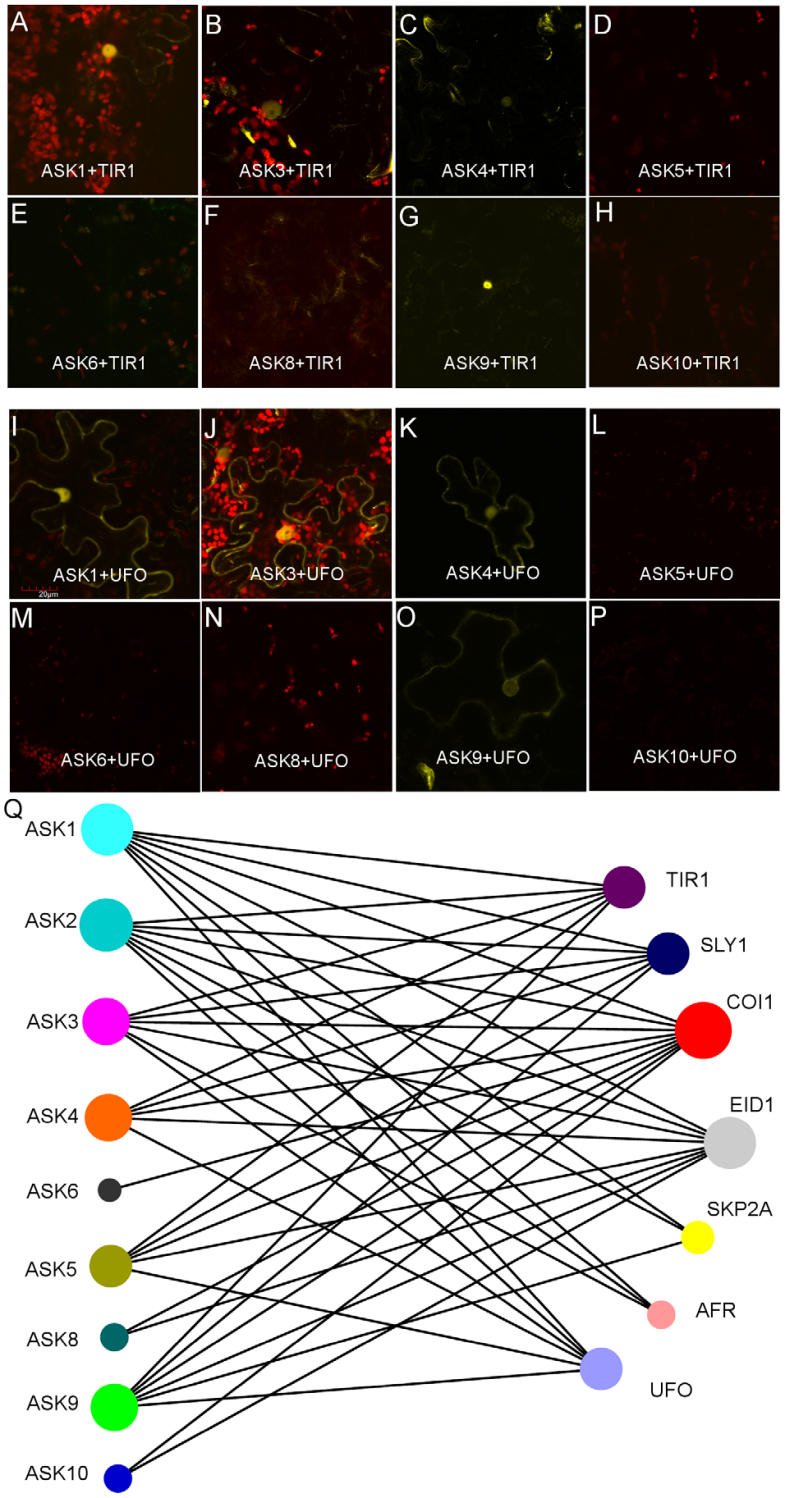
Interaction profile of select ASK and F-Box proteins as assessed using BiFC. Visualization of BiFC-based sub-cellular protein interactions between select ASK and F-Box proteins (TIR1 or UFO) in *N. benthamiana* epidermal leaf cells: YFP signal indicates a positive interaction; chlorophyll auto-fluorescence is shown in red. **A–H**; protein interactions between TIR1 and the ASK1,3,4,9 proteins expressed as BiFC fusion expression constructs. **I–P**; protein interactions between UFO and ASK1,3,4,9 proteins expressed as BiFC fusion expression constructs. **Q;** The indicated interaction map was developed as described in the methods, and summarizes the results obtained following transient expression of BiFC constructs in *N. benthamiana* leaf epidermal cells. Edge lines joining nodes represent a positive interaction.

Previous studies have shown that individual protein products of the *ASK* gene family exhibit both general as well as specific protein-protein interactions involving select F-Box proteins, when assessed in the heterologous yeast 2-hybrid system [21], [50]. This property of generalized versus specific F-box protein interaction capability is of importance for determining which specific ASK polypeptides are capable of interacting with specific F-Box proteins, with implications for the combinatorial diversity of SCF E3-Ub complexes that could form *in vivo* involving subunit polypeptides of either class. Accordingly, we undertook to analyze the *in situ* interaction of polypeptides encoded by *ASK1,2,3,4,5,6,8,9* and *ASK10* in combination with a selected panel of 7 F-Box proteins (TIR1, SLY1, COI1, EID1, SKP2A, AFR and UFO) known to participate in SCF complex assembly. These proteins are involved in the regulation of diverse aspects of plant development such as auxin sensing, circadian rhythm maintenance, ethylene sensing, patterning and development [4], [10], [51]–[59]. Protein interactions were assessed as the reconstitution of fluorescence signal upon co-expression of all 56 pair-wise combinations of split-YFP fusion expression constructs assembled as described in the methods, and subsequently expressed through co-infection of Agrobacterium-containing constructs in the high-fidelity *N. benthamiana* transient expression system [33]. The data for a subset of 16 pair-wise combinations of ASK-F-Box interactions are summarized in Fig. 5 with the exception of ASK2 which is shown in Figure S7.

With respect to protein interactions involving TIR1, five ASK polypeptides (ASK1,2,3,4 and ASK9) were found to interact with TIR1 *in vivo*. Interestingly, the interaction signal observed was predominantly localized to the nucleus in leaf epidermal cells (Fig. 5A–D). Similarly, expression fusion products involving ASK1,2,3,4 and 9 were found to interact with UFO in a pattern that was analogous to TIR1. Although closely related at the primary amino acid sequence level with ASK9, split-YFP expression constructs involving ASK10 did not detectably interact *in vivo* with TIR1 or UFO.

The results summarizing binary protein interactions involving the remaining 40 F-Box-ASK combinations are depicted in the interaction map shown in Fig. 5Q. For all protein interactions involving COI and TIR1, the YFP signal was restricted to the nucleus, whereas all other F-Box-ASK protein combinations were observed in both the cytoplasm and the nucleus. Also noteworthy was the finding that SKP2a interacted with ASK3 in the split-YFP system but not with ASK4, notwithstanding the high degree of deduced primary amino acid sequence similarity between these two ASK polypeptides (94.5%).

Considering that all ASK and F-Box proteins tested were found to interact with at least one other binding partner, we concluded that the observed negative interacting protein combinations were biologically significant and were not due to low levels of protein expression. To confirm that protein expression among the observed negative interacting combinations was comparable to that of positive interacting proteins, we cloned select F-Box and ASK coding domains into a second set of BIFC vectors where the split-YFP coding region incorporated c-Myc- and HA-tag domains, thus facilitating comparative protein expression studies via Western Blot analysis [30]. As shown in Figure S6, from the select proteins investigated all were expressed to levels qualitatively comparable to that of the positive interacting proteins, indicating that the observed negative interactions were not due to the lack of protein expression.

## Discussion

In evolutionary terms, and when compared with other well-characterized multi-cellular model systems, plants exhibit a relatively complex complement of genes that are known or predicted to encode subunit components of the SCF-class of E3-Ub ligase complexes [1], [60]. Despite having one of the smallest known genomes among angiosperm plants [61], Arabidopsis harbours a Skp1-like protein subunit-encoding gene family that is several-fold to orders-of-magnitude greater in complexity than the single gene found in mammals or yeast [39], [62], [63], with variable degrees of complexity found in other model systems such as *Caenorhabditis elegans* (21 genes) or *Drosophila melanogaster* (6 genes) [64], [65]. The apparent high degree of gene duplication of *SKP1* genes is a feature of plants in general (e.g., the 28 *Skp1-*like genes in rice; [39]). Furthermore, the average rate of gene duplication of the *ASK* gene family in comparison to other gene families in Arabidopsis is roughly ten times higher [39]. This feature of plant SCF-E3 ligase subunit-coding complexity can be traced to their earliest evolutionary origin in pre-vascular plants, as revealed by the predicted proteome containing 4 *Skp1*-like genes in the moss, *Physcomitrella patens* [66].

Given the complexity of gene families that are known or predicted to encode subunits of SCF complexes in Arabidopsis, studies ascribing function to individual genes will minimally involve an assessment of subunit functional redundancy, together with an evaluation of the specific SCF complexes that may assemble across developmental time and space. Notions of functional redundancy can be approached from several perspectives, including genetic approaches where loss-of-function alleles in genetic backgrounds carrying single or combinations of mutant loci can be examined for aberrant phenotypes. Single and multiple mutant lines are also valuable for complementation studies from which insight into structure-function relationships involving specific proteins can be derived. Where large gene families are involved, genetic approaches to defining redundancy are commonly guided by phylogeny and clustering of gene products based on their deduced primary amino sequence [67].

Functional redundancy among gene paralogs has been recently extended to include multiple independent biological criteria including quantitative assessment of gene expression, protein-interaction networks involving specific gene products, as well as sub-cellular compartmentation of individual gene products in recognition of the potential complexity of regulatory interactions and functional divergence among genes duplicated in the course of evolution [68]. The analysis presented here embraces this approach, and highlights variations in key functional properties among the Arabidopsis *ASK* gene family.

Clade analysis reveals that clade 1 is comprised of two *ASK* genes, *ASK1* and *ASK2* with an overall similarity of greater than 83% between these two genes. These two genes are also representative of the four *ASK* genes (*ASK1, 2, 3* and *4*) bearing an intron. *ASK1* and *ASK2* are thought to be the evolutionary result of a segmental duplication and subsequent slow evolution of a highly conserved function in plants [39], [43]. Although *ASK1* exhibits a higher transcript level than *ASK2*, the expression pattern of these two genes shows a high degree of co-regulation in terms of transcript abundance. The BiFC-based protein interaction profile described here for ASK1 and ASK2 confirms earlier findings arising from yeast 2-hybrid and co-enrichment experiments that highlight the broad F-Box protein interaction potential of both ASK1 and ASK2 proteins [21]. This pattern of broad interaction potential suggests that both ASK1 and ASK2 participate in a combinatorially diverse array of SCF complexes, which is consistent with the findings from genetic studies that these two proteins exhibit significant functional overlap and are essential for growth early in development [40]. The high degree of similarity in terms of interaction and expression profile is reminiscent of the phylogenetic clades defined by primary deduced amino acid sequence similarity, which could be in part explained by the high level of conservation of these two genes during the course of Arabidopsis evolution.

The second clade is defined by *ASK3* and *ASK4* as two members of the *ASK* gene family whose products are predicted to be highly similar (94.5% at the deduced protein level) and where both genes possess an intron. The high degree of similarity of the deduced protein products of these genes might suggest a similar interaction profile by the two proteins, in a manner reminiscent of ASK1 and ASK2. However, we describe here the finding that ASK3 was able to interact with the SKP2a F-Box protein whereas ASK4 could not in the split-YFP system. Furthermore, the *ASK3* and *ASK4* transcripts exhibited markedly different steady-state abundance where *ASK4* levels were consistently ten-fold higher than *ASK3* in most organ samples. The exceptions were flowers (both stages 9 and 15) and green siliques, where an inverse abundance relationship was observed, characterized by a much-elevated relative abundance of *ASK3* transcripts over those of *ASK4*. The relative high level of expression of *ASK4* might in part be explained by the suggestion that *ASK4* is hypothesized to have arisen as the result of large-scale segmental duplication of the highly expressed *ASK1* [39]. While our studies have not yet been extended to include a corresponding analysis of ASK3 versus ASK4 protein abundance, the available data suggests a significant disparity in gene expression levels between these two close paralogs thus providing a possible explanation for the evolutionary retention of these two genes. In light of our previous finding that ASK3 and ASK4 exhibited similar F-Box interaction profiles in the yeast 2-hybrid system [21], coupled with the finding that both share a common cytoplasmic/nuclear sub-cellular localization in the transient Nicotiana expression system, the data suggest that SCF complexes incorporating ASK4 subunits will be relatively abundant in most foliar organs, roots and whole seedlings, with the exception of late-stage flowers and green siliques where ASK3-containing complexes may preferentially form.

Clade three is comprised of *ASK5* and *ASK6* as two genes which are rapidly evolving and seem to be less conserved throughout plant evolution [39]. This clade includes a controversial member of the *ASK* gene family, *ASK6*, which has been suggested to be a pseudogene due to the extreme low abundance of its transcript in Arabidopsis, coupled with an apparent C-terminal truncation of its deduced polypeptide sequence. Although we likewise could not detect transcripts for ASK6 in the organs examined, others have nevertheless managed to clone a full-length *ASK6* cDNA as part of the high-throughput cDNA cloning of low-expressing genes (C. Town, J. Craig Venter Institute, personal communication). As described here, the ASK6 protein containing an extended coding region was found to interact exclusively with COI1, suggesting a role for ASK6 in some aspect of jasmonate signal generation or perception. We conclude that ASK6 is both expressed and is functional at the polypeptide level. The data presented in this study show that both *ASK5* and *ASK6* have divergent expression profiles and their corresponding proteins exhibit different interaction profiles despite their high degree of similarity at the deduced protein level (∼70%). As described here, the *ASK5* transcript abundance more closely parallels that of *ASK14* in various organ samples in a way that was not predicted by sequence-based phylogeny.

The *ASK7,8,9,10* clade is defined by a group of closely related genes that are also tightly clustered within a 10 Kbp span of chromosome 3 (Figure S1), suggesting a recent localized tandem duplication event and a possible conserved expression profile for these genes. Indeed hierarchical clustering of transcript expression resulted in a relatively tight clustering of these genes reminiscent of their phylogenetic clade. Strikingly, a distinct protein interaction profile was observed among the genes within this cluster, where ASK8 and ASK10 interacted solely with COI1 and EID1, whereas ASK9 interacted more generally with 6 out of the 7 F-Box proteins tested with the exception of AFR. Taken together, the results suggest a strong sub-functionalization and a possible basis for evolutionary retention of the four genes within this clade.

The clade defined by *ASK14,15,16,17,18,19* appears to have diverged from *ASK3* and *ASK4* as a result of an initial retrotransposition event followed by two recent tandem duplications within the clade, giving rise to the 5 clade members [39]. Given the probable recent evolution of the clade, one might anticipate similar expression and protein interaction profiles among its members. Although we did not investigate the sub-cellular localization or protein interaction properties of genes in this clade, the expression profile of the genes was found to exhibit a semi-divergent expression profile where *ASK14* and *ASK16* exhibited an expression profile distinct from the other genes in this clade, but highly similar to that of *ASK5* and *ASK13*, respectively. Interestingly *ASK16* and *ASK17,* with close to 62% protein sequence similarity, also have a high similarity expression profile, with a minor difference in stage 9 flowers where *ASK16* transcripts were expressed with no detectable expression of *ASK17*.

Defining nodes of *ASK* gene expression and ASK protein interaction at a higher resolution across different spatial and temporal dimensions at the organ, cellular and sub-cellular level, may contribute to an improved understanding of potential function, or at least lead to the advancement of predictive hypotheses for the formation of specific SCF complexes with an inferred biological function. An illustrative example arises from the selective interaction of ASK6 with COI, where a role for jasmonate signalling is inferred among other possible functional roles for this member of the ASK protein family. Similarly, the interaction of ASK10 with EID1 may suggest that this member of the ASK protein family engages in the formation of a subset of SCF complexes that play a role in ethylene signal perception in Arabidopsis. Recent findings have shown that FBL17, an F-box protein which is known to be involved in cell cycle regulation during male gametogenesis, is able to interact with ASK11 in yeast 2-hybrid and BiFC-based studies [69], which is in full accord with our observation of a flower-specific expression pattern for *ASK11.*

The study presented here serves to highlight the importance of defining functional equivalence or redundancy from multiple biological perspectives, here including quantitative gene expression profiling, sub-cellular localization and *in vivo* analysis of protein interaction potential. A third perspective arises from the phenotypic analysis of individual loss-of-function alleles, both alone and in combination. A genetic perspective of redundancy within the *ASK* gene family is complicated by the fact that genes in this family are generally small and are therefore significantly under-represented for null alleles within reverse-genetic resources such as the T-DNA insertion mutagenesis population organized as the SIGnAL resource [22]. Future work to define genetic interactions within the *ASK* gene family would logically involve transgenic miRNA expression interdiction approaches [70], the development of alternative reverse genetic resources [71], or perhaps targeted deletion strategies directed to the disruption of *ASK* gene function [72].

In the present study, we have characterized the steady-state transcript levels, sub-cellular distribution profiles, together with the analysis of a restricted set of protein-protein interactions within the confines of a set of ‘standard’ laboratory propagation and environmental interaction conditions. It remains to be seen whether the observed expression, localization and gene product interaction profiles described here might further diversify in the face of changing environmental conditions, or treatment with different plant growth regulatory compounds with which several of the proteins involved are directly implicated. Indeed, related studies suggest that sub-cellular localization of select ASK-F-Box protein interactions with a known role in hormone signal perception are altered in the presence of the relevant signalling compounds, thus presenting an additional dimension for our understanding of the molecular basis of their function (Dezfulian *et al.*, manuscript in preparation).

Notwithstanding its relatively small genome size and genetic complexity, Arabidopsis has nevertheless retained a complex family of closely related genes encoding Skp1-like proteins. This observation, which can be extrapolated more generally to other model plant species for which significant genome content information is available, is a general feature of plants and may relate to their unique adaptive strategy as sessile organisms in dynamic interaction with their environment. In basic terms, the large number of structurally related genes raises questions surrounding the issue of functional redundancy versus evolutionary retention within the gene family. In this study, we provide some novel perspectives on the issue of redundancy which may help to explain the retention of these genes during Arabidopsis genome evolution. Although we cannot currently rule out the possibility that the other *ASK* genes not investigated as part of this study show unique sub-cellular localization, the similar localization pattern observed for the all the ASK proteins studied suggests that there has been no evolutionary pressure for retention arising from specialized sub-cellular localization among *ASK* gene products. Our finding that structurally closely-related genes are nonetheless distinguished on the basis of expression and/or protein interaction profiles offers an alternative explanation for their retention based on functional diversification. Indeed, virtually every clade defining the phylogeny of the ASK protein family was found to contain one or more members that have diverged at the level of expression and/or protein interaction profile, thus defining a potential molecular basis for an expanded functional repertoire. Only through an expanded study that includes additional facets of redundancy will a reliable picture of functional redundancy within the *ASK* gene family emerge, with implications for future studies into their shared versus divergent molecular functions.

## Supporting Information

Figure S1 **ASK Gene Chromosomal Location.** Chromosomal location of the *ASK* gene family in the genome of Arabidopsis.
(TIF)

Figure S2 **Confocal imaging and sub-cellular localization of CFP fusion proteins in** ***N. benthamiana.*** C-terminal CFP fusion proteins were transiently expressed in *N. benthamiana* leaf epidermal cells and visualized using confocal microscopy. **A**; TIR:CFP fusion protein. **B,C**; CUL1:CFP and ASK1:CFP fusion protein. The sub-cellular localization of ASK1:CFP in *N. benthamiana* leaves parallels that of YFP:ASK1 in transgenic Arabidopsis lines, and confirms the fidelity of the *N. benthamiana* transient expression assay.
(TIF)

Figure S3 **Hierarchical Clustering of** ***ASK*** **Gene Expression.** Hierarchical clustering of publically available microarray expression data for *ASK* genes across different Arabidopsis tissues, using the Expression Browser tool found online at http://bar.utoronto.ca. The difference observed between this clustering and that generated by the present study can be attributed principally to the non-uniqueness of the probes used in construction of the microarrays, coupled with the higher resolution of the qRT-PCR data.
(TIF)

Figure S4 **Expression and localization of YFP-ASK8 fusion protein in transgenic Arabidopsis. A**; Localization of ASK8:YFP in leaves and roots of transgenic plants. The YFP:ASK8 fusion protein was found to aggregate exclusively in the leaves of transgenic plants, but exhibited a similar pattern to that of other YFP:ASK fusion proteins in the roots of the same transgenic plants. **B**; Comparison of YFP:ASK8 and YFP:ASK1 protein expression in three different transgenic Arabidopsis lines, where YFP:ASK1 expression showed no sign of aggregation. The results indicate that the observed signal aggregation in the ASK8:YFP transgenic backgrounds were not due to over-expression of the fusion protein.
(TIF)

Figure S5 **Confocal imaging and sub-cellular localization of YFP:ASK protein fusions in transgenic Arabidopsis.** Fusion protein visualization in stable transgenic lines was carried out as described in the methods. **A**,**B**,**C**,**E**,**G**, **J**; sub-cellular localization of YFP:ASK1, YFP:ASK2, YFP:ASK4, YFP:ASK8 and YFP:ASK10 in root tissues, respectively. **D**,**F**,**H**,**I**; localization of YFP:ASK5, YFP:ASK8, YFP:ASK9 and YFP:ASK10 in leaf tissues, respectively.
(TIF)

Figure S6 **Protein expression verification of the split-YFP fragments in the BiFC assay.** Following visualization of the BIFC signal, injected *N. benthamiana* leaves were subjected to protein extraction and immunoblotting (IB), the expression of ASK1, ASK3, ASK6 and ASK8 in combination with TIR1, SKP2A, SLY1 and AFR was examined. The ASK genes were cloned into a Myc-tag BiFC vector and the F-Box proteins in an HA-tagged BiFC vector. Protein immunoblots decorated with anti-Myc (left section) and anti-HA (right section) antibodies were used for detection of the nEYFP:ASK, and cEYFP:F-Box fusion proteins, respectively.
(TIF)

Figure S7 **Sub-cellular localization of BiFC Signals.** The sub-cellular localization of BIFC signals were assessed by determining co-localization of select BiFC signals with the nuclear-specific propidium iodide (PI) signal, as described. **A**,**B**,**C**,**D**,**E**; the YFP fluorescent signal from the BiFC assays. **F**,**G**,**H**,**I**,**J**; fluorescent signal from the PI-stained Nuclei.
(TIF)

Table S1 Gene Names and locus identifiers for genes used in this study.
(DOC)

Table S2 Primers used for stop codon removal in Gateway Vectors.
(DOC)

Table S3 Plasmid Constructs Generated by the Study.
(DOC)

Table S4 Primers used for stop codon removal in Gateway Vectors.
(DOC)

Table S5 Normalized qRT-PCR values.
(XLSX)
