# Supplementary figures and images for: The SKP1-Like Gene Family of Arabidopsis Exhibits a High Degree of Differential Gene Expression and Gene Product Interaction during Development
Source: PLoS One. 2012 Nov 30;7(11):e50984. doi: 10.1371/journal.pone.0050984 (PMC3511428; doi:10.1371/journal.pone.0050984)

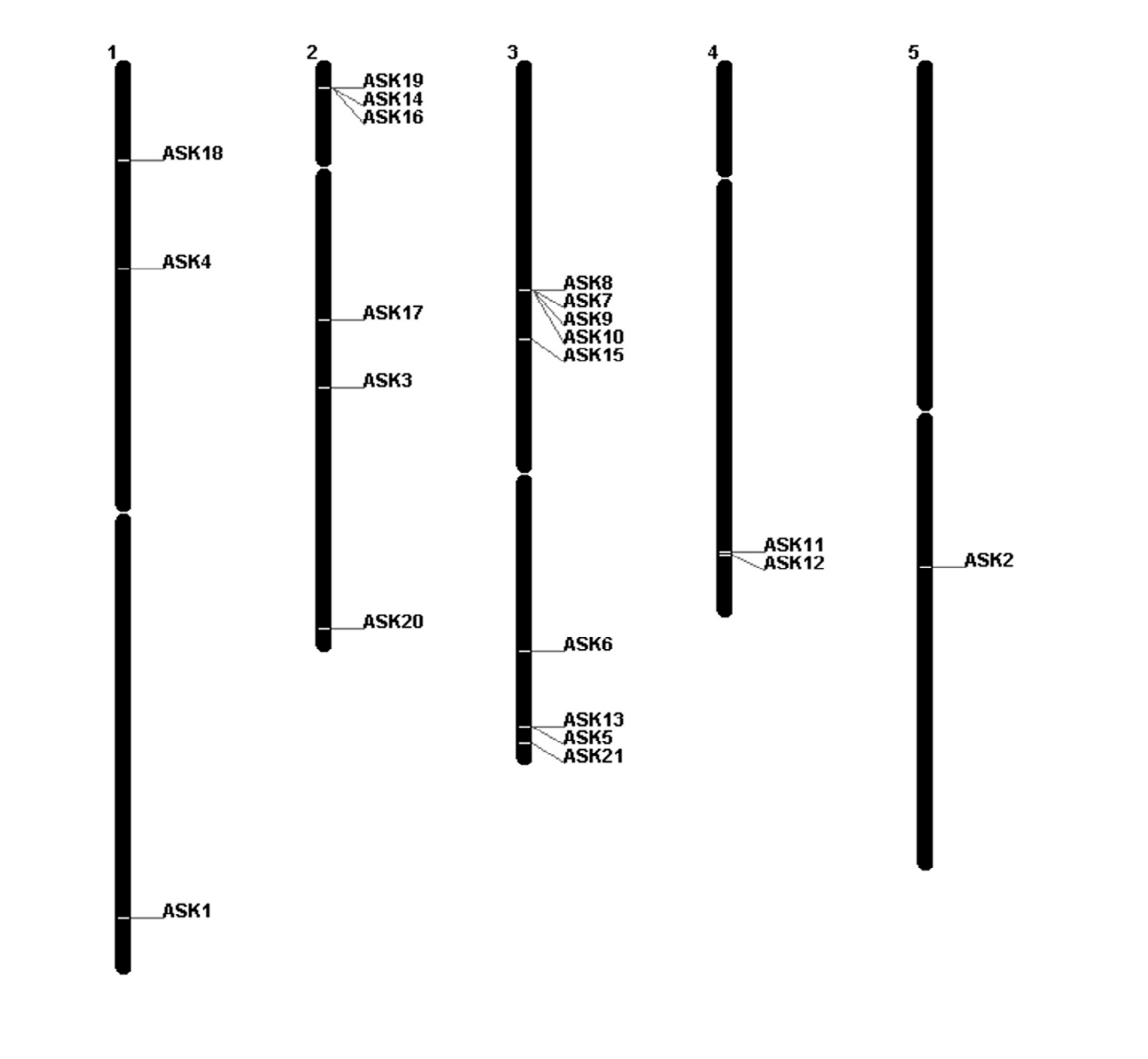

Supplement: Figure S1 — ASK Gene Chromosomal Location. Chromosomal location of the ASK gene family in the genome of Arabidopsis. (TIF) [file pone.0050984.s001.tif]

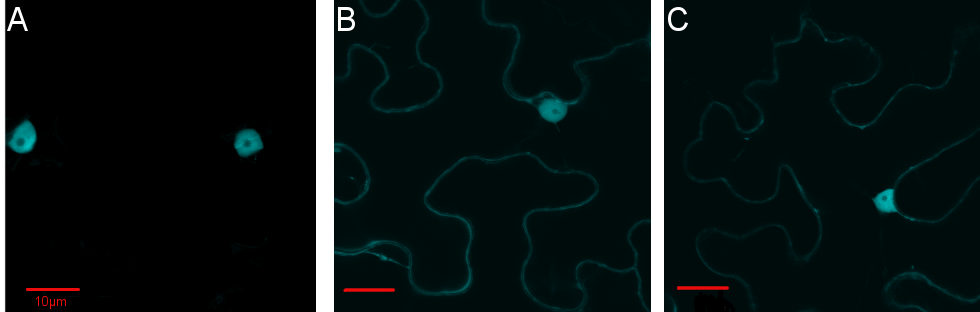

Supplement: Figure S2 — Confocal imaging and sub-cellular localization of CFP fusion proteins in N. benthamiana. C-terminal CFP fusion proteins were transiently expressed in N. benthamiana leaf epidermal cells and visualized using confocal microscopy. A; TIR:CFP fusion protein. B,C; CUL1:CFP and ASK1:CFP fusion protein. The sub-cellular localization of ASK1:CFP in N. benthamiana leaves parallels that of YFP:ASK1 in transgenic Arabidopsis lines, and confirms the fidelity of the N. benthamiana transient expression assay. (TIF) [file pone.0050984.s002.tif]

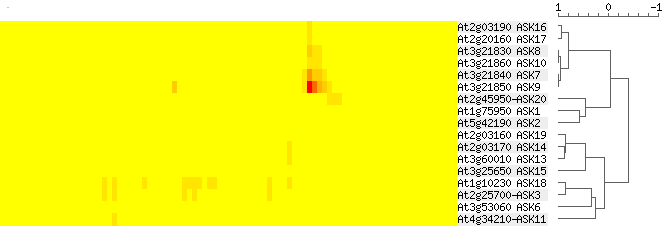

Supplement: Figure S3 — Hierarchical Clustering of ASK Gene Expression. Hierarchical clustering of publically available microarray expression data for ASK genes across different Arabidopsis tissues, using the Expression Browser tool found online at http://bar.utoronto.ca. The difference observed between this clustering and that generated by the present study can be attributed principally to the non-uniqueness of the probes used in construction of the microarrays, coupled with the higher resolution of the qRT-PCR data. (TIF) [file pone.0050984.s003.tif]

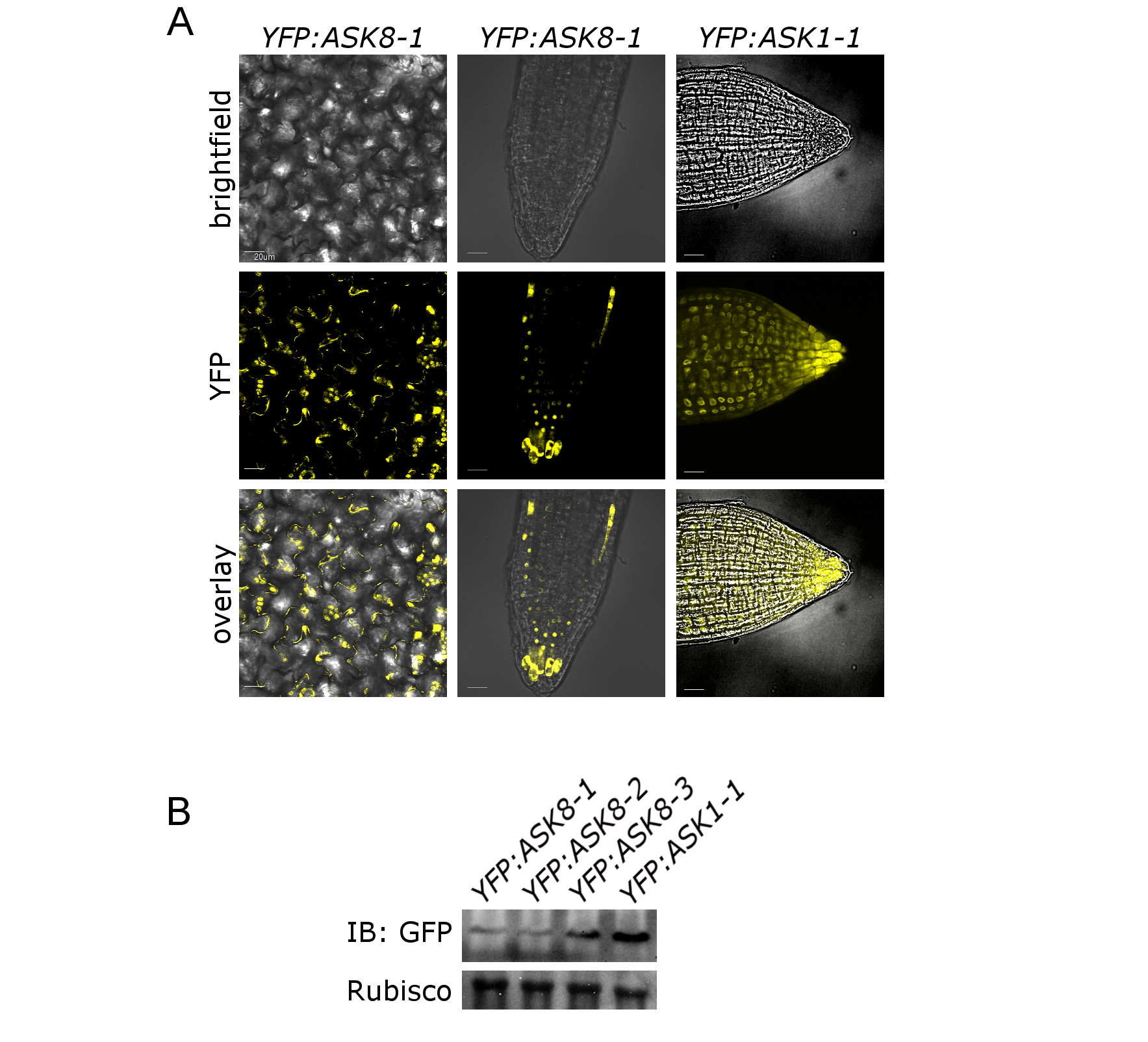

Supplement: Figure S4 — Expression and localization of YFP-ASK8 fusion protein in transgenic Arabidopsis. A; Localization of ASK8:YFP in leaves and roots of transgenic plants. The YFP:ASK8 fusion protein was found to aggregate exclusively in the leaves of transgenic plants, but exhibited a similar pattern to that of other YFP:ASK fusion proteins in the roots of the same transgenic plants. B; Comparison of YFP:ASK8 and YFP:ASK1 protein expression in three different transgenic Arabidopsis lines, where YFP:ASK1 expression showed no sign of aggregation. The results indicate that the observed signal aggregation in the ASK8:YFP transgenic backgrounds were not due to over-expression of the fusion protein. (TIF) [file pone.0050984.s004.tif]

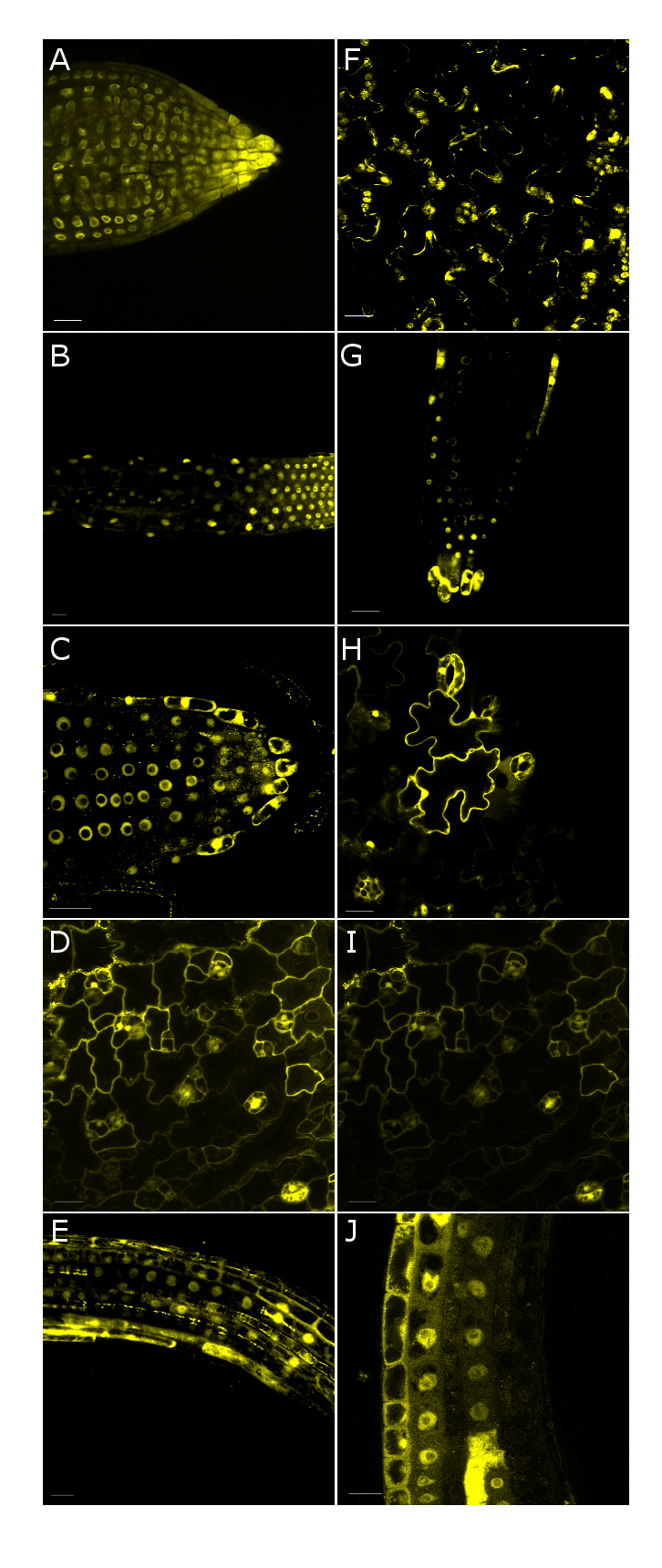

Supplement: Figure S5 — Confocal imaging and sub-cellular localization of YFP:ASK protein fusions in transgenic Arabidopsis. Fusion protein visualization in stable transgenic lines was carried out as described in the methods. A,B,C,E,G, J; sub-cellular localization of YFP:ASK1, YFP:ASK2, YFP:ASK4, YFP:ASK8 and YFP:ASK10 in root tissues, respectively. D,F,H,I; localization of YFP:ASK5, YFP:ASK8, YFP:ASK9 and YFP:ASK10 in leaf tissues, respectively. (TIF) [file pone.0050984.s005.tif]

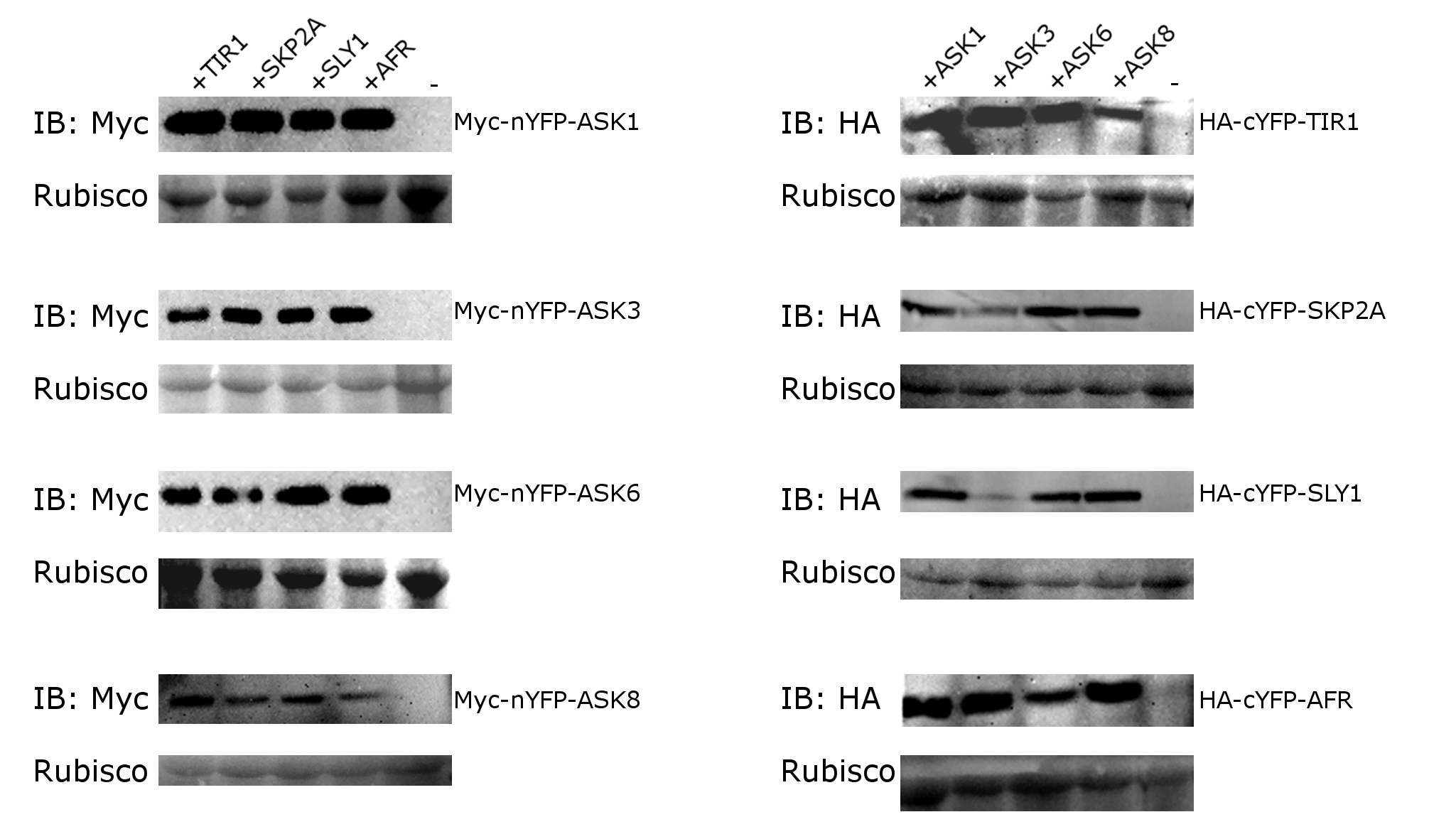

Supplement: Figure S6 — Protein expression verification of the split-YFP fragments in the BiFC assay. Following visualization of the BIFC signal, injected N. benthamiana leaves were subjected to protein extraction and immunoblotting (IB), the expression of ASK1, ASK3, ASK6 and ASK8 in combination with TIR1, SKP2A, SLY1 and AFR was examined. The ASK genes were cloned into a Myc-tag BiFC vector and the F-Box proteins in an HA-tagged BiFC vector. Protein immunoblots decorated with anti-Myc (left section) and anti-HA (right section) antibodies were used for detection of the nEYFP:ASK, and cEYFP:F-Box fusion proteins, respectively. (TIF) [file pone.0050984.s006.tif]

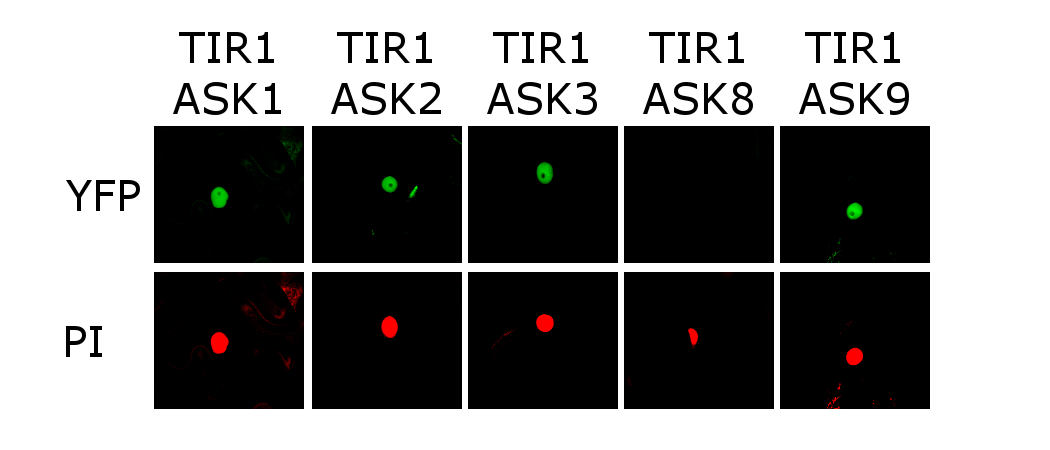

Supplement: Figure S7 — Sub-cellular localization of BiFC Signals. The sub-cellular localization of BIFC signals were assessed by determining co-localization of select BiFC signals with the nuclear-specific propidium iodide (PI) signal, as described. A,B,C,D,E; the YFP fluorescent signal from the BiFC assays. F,G,H,I,J; fluorescent signal from the PI-stained Nuclei. (TIF) [file pone.0050984.s007.tif]
