# Supplementary material for: The SKP1-Like Gene Family of Arabidopsis Exhibits a High Degree of Differential Gene Expression and Gene Product Interaction during Development
Source: PLoS One. 2012 Nov 30;7(11):e50984. doi: 10.1371/journal.pone.0050984 (PMC3511428; doi:10.1371/journal.pone.0050984)
Supplement: Table S1 — Gene Names and locus identifiers for genes used in this study. (DOC) [file pone.0050984.s008.doc]

**Table S1. Gene Names and locus identifiers for genes used in this study**

| **Gene Name** | **TAIR Locus identifier** | **ABRC cDNA stock Number** |
| --- | --- | --- |
| *ASK1* | *At1g75950* | G10618 |
| *ASK2* | *At5g42190* | N/A |
| *ASK3* | *At2g25700* | G13779 |
| *ASK4* | *At1g20140* | G14059 |
| *ASK5* | *At3g60020* | PENTR221-AT3G60020 |
| *ASK6* | *At3g53060* | PENTR221-AT3G53060 |
| *ASK7* | *At3g21840* | N/A |
| *ASK8* | *At3g21830* | PENTR221-AT3G21830 |
| *ASK9* | *At3g21850* | PENTR221-AT3G21850 |
| *ASK10* | *At3g21860* | PENTR221-AT3G21860 |
| *ASK11* | *At4g34210* | PENTR221-AT4G34210 |
| *ASK12* | *At4g34470* | N/A |
| *ASK13* | *At3g60010* | N/A |
| *ASK14* | *At2g03170* | PENTR221-AT2G03170 |
| *ASK15* | *At3g25650* | PENTR221-AT3G25650 |
| *ASK16* | *At2g03190* | N/A |
| *ASK17* | *At2g20160* | N/A |
| *ASK18* | *At1g10230* | N/A |
| *ASK19* | *At2g03160* | PENTR221-AT2G03160 |
| *ASK20* | *At2g45950* | N/A |
| *ASK21* | *At3g61415* | N/A |
| *CUL1* | *At4g02570* | G09998 |
| *TIR1* | *At3g62980* | GC105370 |
| *AFR* | *At2g24540* | G21324 |
| *COI1* | *At2g39940* | G12955 |
| *EID1* | *At4g02440* | G15754 |
| *SKP2A* | *At1g21410* | G14226 |
| *SLY1* | *At4g24210* | G50138 |
| *UFO* | *At1g30950* | PENTR221-AT1G30950 |
