# Supplementary material for: The SKP1-Like Gene Family of Arabidopsis Exhibits a High Degree of Differential Gene Expression and Gene Product Interaction during Development
Source: PLoS One. 2012 Nov 30;7(11):e50984. doi: 10.1371/journal.pone.0050984 (PMC3511428; doi:10.1371/journal.pone.0050984)
Supplement: Table S3 — Plasmid Constructs Generated by the Study. (DOC) [file pone.0050984.s010.doc]

#### Table S3. Plasmid Constructs Generated by the Study

| pENTR | pDestination | pExpression |
| --- | --- | --- |
| ASK1 | pEarlygate104(35S-YFP-attR) | 35S-YFP-ASK1 |
| ASK3 | pEarlygate104(35S-YFP-attR) | 35S-YFP-ASK3 |
| ASK4  ASK5  ASK6 | pEarlygate104(35S-YFP-attR)  pEarlygate104(35S-YFP-attR)  pEarlygate104(35S-YFP-attR) | 35S-YFP-ASK4  35S-YFP-ASK5  35S-YFP-ASK6 |
| ASK8 | pEarlygate104(35S-YFP-attR) | 35S-YFP-ASK8 |
| ASK9 | pEarlygate104(35S-YFP-attR) | 35S-YFP-ASK9 |
| ASK10 | pEarlygate104(35S-YFP-attR) | 35S-YFP-ASK10 |
| ASK1 | pEarlygate102(35S-attR-CFP) | 35S-ASK1-CFP |
| TIR1 | pEarlygate102(35S-attR-CFP) | 35S-TIR1-CFP |
| CUL1 | pEarlygate102(35S-attR-CFP) | 35S-CUL1-CFP |
| ASK1 | BiFP3(35S-cYFP-attR) | 35S-cYFP-ASK1 |
| ASK3 | BiFP3(35S-cYFP-attR) | 35S-cYFP-ASK3 |
| ASK4 | BiFP3(35S-cYFP-attR) | 35S-cYFP-ASK4 |
| ASK5 | BiFP3(35S-cYFP-attR) | 35S-cYFP-ASK5 |
| ASK6 | BiFP3(35S-cYFP-attR) | 35S-cYFP-ASK6 |
| ASK8 | BiFP3(35S-cYFP-attR) | 35S-cYFP-ASK8 |
| ASK9 | BiFP3(35S-cYFP-attR) | 35S-cYFP-ASK9 |
| ASK10 | BiFP3(35S-cYFP-attR) | 35S-cYFP-ASK10 |
| UFO | BiFP2(35S-nYFP-attR) | 35S-nYFP-UFO |
| TIR1 | BiFP2(35S-nYFP-attR) | 35S-nYFP-TIR1 |
| COI1 | BiFP2(35S-nYFP-attR) | 35S-nYFP-COI1 |
| EID1 | BiFP2(35S-nYFP-attR) | 35S-nYFP-EID1 |
| AFR | BiFP2(35S-nYFP-attR) | 35S-nYFP-AFR |
| SLY1 | BiFP2(35S-nYFP-attR) | 35S-nYFP-SLY1 |
| SKP2A | BiFP2(35S-nYFP-attR) | 35S-nYFP-SKP2 |
