# Supplementary material for: The SKP1-Like Gene Family of Arabidopsis Exhibits a High Degree of Differential Gene Expression and Gene Product Interaction during Development
Source: PLoS One. 2012 Nov 30;7(11):e50984. doi: 10.1371/journal.pone.0050984 (PMC3511428; doi:10.1371/journal.pone.0050984)
Supplement: Table S4 — Primers used for stop codon removal in Gateway Vectors. (DOC) [file pone.0050984.s011.doc]

**Table S4. Primers used for stop codon removal in Gateway Vectors**

All primers are indicated in the 5’ – 3’ orientation.

| attB1-AtCUL1 | GGGGACAAGTTTGTACAAAAAAGCAGGCTTAATGCCAACTTTGTACAAAAAAG |
| --- | --- |
| attB2-AtCUL1-stop | GGGGACCACTTTGTACAAGAAAGCTGGGTAAGCCAAGTACCTAAACATGTTA |
| attB1-AtTIR1 | GGGGACAAGTTTGTACAAAAAAGCAGGCTTAATGCCAACTTTGTACAAAAAAG |
| attB2-AtTIR1-stop | GGGGACCACTTTGTACAAGAAAGCTGGGTATAATCCGTTAGTAGTAATGATT |
| attB1-AtASK1 | GGGGACAAGTTTGTACAAAAAAGCAGGCTTAATGTCTGCGAAGAAGATTGTGT |
| attB2-AtASK1-stop | GGGGACCACTTTGTACAAGAAAGCTGGGTATTCAAAAGCCCATTGGTTCTCT |
